# Pancréas ectopique de localisation hépatique

**DOI:** 10.11604/pamj.2020.36.250.21309

**Published:** 2020-08-06

**Authors:** Faten Limaïem

**Affiliations:** 1 Université de Tunis El Manar, Faculté de Médecine de Tunis, Tunis, Tunisie

**Keywords:** Pancréas ectopique, foie, anatomie pathologique, Ectopic pancreas, liver, pathological anatomy

## Abstract

Ectopic pancreas (or aberrant or pancreatic heterotopia) is defined as pancreatic tissue outside the boundaries of the pancreas, without anatomic connection to the main gland. Its incidence is between 0.5% and 14% in autopsy series, but it is probably underestimated because it is often asymptomatic. It most commonly affects the duodenum (30-35%), the stomach (30%), and the jejunum (15%). Hepatic involvement is exceptional. We here report a new case of unexpected detection. The study involved a 54-year-old, hypertense female patient who had undergone surgery for adenocarcinoma of the sigmoid classified pT3N1b (A) two years before. Disease progression was marked by the occurrence of multiple predominant secondary nodules in the right hepatic lobe (B). Neoadjuvant chemotherapy (4 cicles of FOLFOX-ERBITUX) was started. The patient received right lobectomy. The histological examination of the different surgical specimens confirmed the presence of secondary, moderately differentiated hepatic adenocarcinoma of colorectal origin with healthy surgical excision margins. The surrounding liver was the site of unsystematized macrovacuolar steatosis in 20% of cases. It was associated with a focus of pancreatic heterotopia, including some acini and excretory canals arranged in lobules (C, D). The postoperative course was simple; however, patient’s outcome was marked by recurrence of cancer in the left lobe of the liver.

## Image en médecine

Le pancréas ectopique (ou aberrant ou hétérotopie pancréatique) se définit par la présence de tissu pancréatique en situation anormale, sans rapport anatomique avec la glande principale. Son incidence est estimée entre 0,5% et 14% sur les séries autopsiques, mais est probablement sous-estimée du fait de son caractère le plus souvent asymptomatique. Les localisations habituelles sont représentées par le duodénum (30-35%), l´estomac (30%) et le jéjunum (15%). Sa localisation hépatique est exceptionnelle. Nous en rapportons un nouveau cas de découverte fortuite. Il s´agit d´une patiente âgée de 54 ans, hypertendue, opérée deux ans auparavant pour un adénocarcinome du sigmoïde classé pT3N1b (A). L´évolution était marquée par l´installation de multiples nodules d´allure secondaire prédominant au niveau du lobe hépatique droit (B), d´où l´instauration d´une chimiothérapie néo-adjuvante (4 cures FOLFOX-ERBITUX). La patiente a bénéficié d´une lobectomie droite. L´examen histologique des différents prélèvements effectués a confirmé la présence d´une localisation secondaire hépatique d´un adénocarcinome moyennement différencié d´origine colorectale avec des limites d´exérèses saines. Le foie alentour était le siège d´une stéatose macrovacuolaire non systématisée estimée à 20%. Il s´y associait un foyer d´hétérotopie pancréatique englobant des acini et des canaux excréteurs organisés en lobule (C, D). Les suites opératoires immédiates étaient simples, cependant, l´évolution a été marquée par la récidive tumorale au niveau du foie gauche.

**Figure 1:**
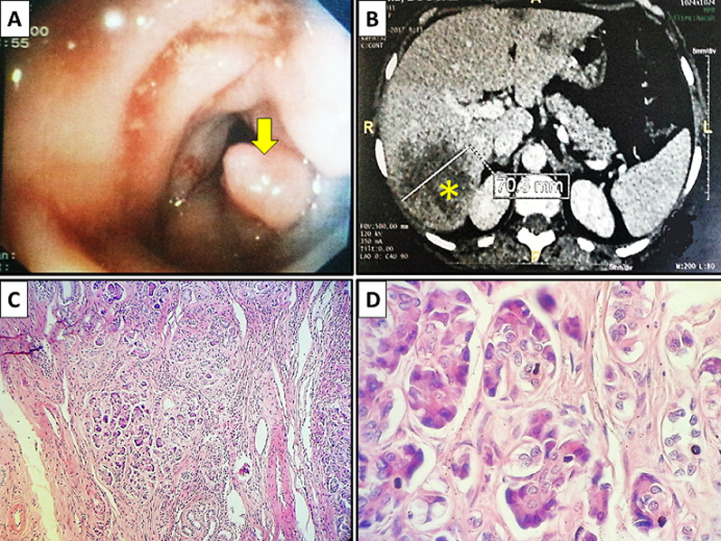
A) aspect endoscopique d'une tumeur bourgeonnante du colon sigmoïde (flèche); B) tomodensitométrie abdominale montrant un foie siège de multiples nodules et masses diffus prédominant au niveau du secteur latéral droit de taille variable entre 0,6 et 7cm (astérisque); C) lobule pancréatique ectopique au niveau du parenchyme hépatique (flèche); (hématoxyline et éosine, × 200); D) acini pancréatiques et canaux excréteurs dépourvus d'atypies cytonucléaires; (hématoxyline et éosine, × 400)

